# Excess mortality related to high air temperature: Comparison of the periods including 1994 and 2018, the worst heat waves in the history of South Korea

**DOI:** 10.1371/journal.pone.0310797

**Published:** 2024-11-13

**Authors:** Jonghyuk Choi, Hyungryul Lim, Sanghyuk Bae, Kyung-Hwa Choi, Xue Han, Mina Ha, Ho-Jang Kwon

**Affiliations:** 1 Department of Preventive Medicine, College of Medicine, Dankook University, Cheonan, Republic of Korea; 2 Research Institute of Healthcare Bigdata, College of Medicine, Dankook University, Cheonan, Republic of Korea; 3 Department of Preventive Medicine and Public Health, Ajou University School of Medicine, Suwon, Republic of Korea; 4 Department of Preventive Medicine, College of Medicine, The Catholic University of Korea, Seoul, Republic of Korea; University of Rajshahi, BANGLADESH

## Abstract

Climate change has caused extreme weather events, including frequent summer heat waves. We examined how the effects of high air temperatures on mortality have changed between the two study periods (1991–1995 and 2015–2019), including 1994 and 2018, the worst heat wave years in the meteorological history of South Korea. Temperature data from the Korea Meteorological Administration and mortality data from Statistics Korea were used in this study. We used distributed lag nonlinear models to estimate the cumulative relative risks (CRRs) to determine the association between daily maximum temperature in summer (June to September) and mortality. CRRs were estimated for each province and pooled using a random-effects meta-analysis for all provinces. Maximum temperature and annual average days in heat wave were 37.7°C and 11.8 in 1991–1995 and 38.3°C and 18.8 in 2015–2019. The slope of the CRR for mortality increases with increasing temperature and has been steeper in the past than in recent years and steeper in those over 65 than in those under 65. Excess mortality has recently declined compared with that in the past. The impact of high summer temperatures on mortality changed between the two periods, suggesting improved population resilience.

## Introduction

Since the industrial era, the world has experienced a steady increase in greenhouse gases such as carbon dioxide, methane, and nitrogen dioxide due to population and economic growth [1]. This surge has resulted in a global temperature rise of approximately 1°C since the pre-industrial era. If the current trajectory persists, it is projected to reach 1.5°C between 2030 and 2052 [2]. Owing to continued global warming, the frequency and intensity of heat waves are expected to increase [2].

The Institute for Health Metrics and Evaluation (IHME), as part of the Global Burden of Disease 2019 project, estimated that there are approximately 308,000 deaths worldwide due to high temperatures [3]. Studies that examined multiple cities in different countries also found an association between high temperatures and all-cause mortality [4–6]. A meta-analysis that evaluated 14 studies conducted in Korea reported that deaths from all causes, cardiovascular disease, respiratory disease, and cerebrovascular disease increased by 5%, 6%, 2%, and 4%, respectively, for every 1°C increase in temperature during the summer season [7]. The effects of high temperatures on mortality can vary depending on characteristics of the subjects such as gender, age, socioeconomic level, spatial characteristics such as countries and cities, and time periods [7–10]. The impact of these rising temperatures on public health, particularly mortality, requires a focused analysis in the context of changing societal and environmental conditions.

The history of South Korea is highlighted by two significant years, 1994 and 2018, which were both marked by severe heat waves. These years are emblematic of major climatic events and act as reference points to investigate the interaction of environmental, infrastructural, and societal changes and their effect on mortality. In a study conducted from 1991 to 2011, the highest number of heat-related deaths was 92 in 1994 [11]. A previous study in Korea estimated that 3,384 additional deaths occurred during the summer of 1994 [12]. According to the Korean Centers for Disease Control and Prevention’s emergency room surveillance system for heat-related illnesses, 4,526 heat-related illnesses and 48 deaths were reported in Korea in 2018, which is the highest level reported recently [13]. According to the Korea Meteorological Administration (KMA), there number of heat wave days was 29.6 in 1994 and 31 in 2018 [14]. In 1994 and 2018, the Tibetan high-pressure area in the upper atmosphere around Korea and the North Pacific high-pressure area in the middle and lower layers of the atmosphere developed more than normal, resulting in hot and humid air. When the weather is clear, there is a strong solar effect; therefore, hot weather persists [15].

The years 1994 and 2018 were highlighted as periods when East Asia, including Korea, Japan, and China, experienced record-breaking severe heat waves [16, 17]. Despite the extreme heat waves that South Korea experienced in 1994 and 2018, there remains a discernible gap in research comparing the effects of these two major heat wave periods in South Korea’s history. Our choice of specific years and periods reflected an effort to understand the impact of these exceptional climatic events on mortality in a rapidly changing societal context. Focusing on these two specific periods allowed us to elucidate how these changes between the two periods have affected deaths related to extreme heat in South Korea. These particular years are critical due to the significant heat wave events they represent in the history of South Korea. They provide a unique lens through which we can examine the interplay between environmental, infrastructural, and societal changes and their impact on heat-related mortality.

Through this focused comparison, this study aimed to reveal the nuanced temporal dynamics of how societal evolution in South Korea influenced the public health outcomes of these extreme climatic events. Therefore, this study focused on and compared the past and recent periods (1991–1995 and 2015–2019) covering 1994 and 2018 by calculating the excess deaths attributable to high temperatures in South Korea.

## Materials and methods

### Study population and regions

**Fig 1:**
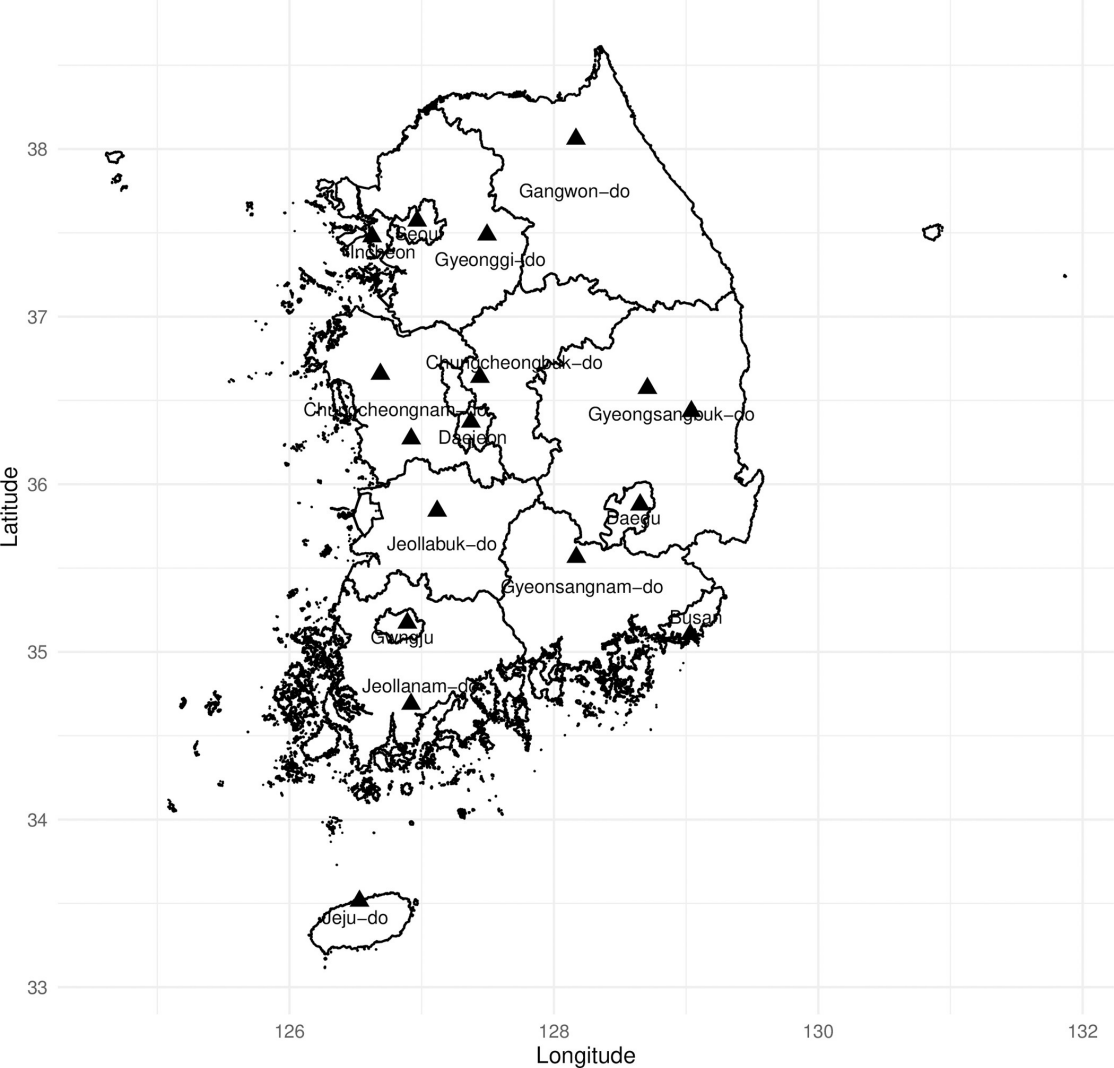
Map of the study area and location of weather stations.

South Korea is a country in East Asia located in the southern half of the Korean Peninsula (33°–38° north latitude and 126°–132° east longitude), with an area of 100,410 km^2^ and a population of approximately 51.6 million in 2022 [18]. The study population comprised the entire Korean population and regions, with the exception of Ulsan Metropolitan City and Sejong Special City, owing to a lack of meteorological data for the period–1991–1995. Fifteen provinces were included: Seoul, Busan, Daegu, Incheon, Gwangju, Daejeon, Gyeonggi-do, Gangwon-do, Chungcheongbuk-do, Chungcheongnam-do, Jeollabuk-do, Jeollanam-do, Gyeongsangbuk-do, Gyeongsangnam-do, and Jeju-do (Fig 1).

### Weather data

To compare the effects of high air temperatures in the past and recent periods, we set each five-year period (1991–1995 and 2015–2019), including 1994 and 2018, which were the worst heat wave years in the meteorological history of South Korea. Only summer (from June to September) of each study year was considered. Data for 1991–1995 and 2015–2019 published by each monitoring station on the KMA’s Open MET Data Portal website were used for meteorological variables, such as daily maximum temperature and relative humidity [14]. The observed meteorological data with the smallest distance between the central coordinates of each province and the coordinates of the monitoring station were used to assign exposure values to each province. In the two periods of 1991–1995 and 2015–2019, of the total 73 and 95 monitoring stations, 15 and 16 monitoring stations were assigned to each provincial daily unit, respectively (Fig 1; In 2015, the weather station in Daegu was terminated and reassigned to a different station ID). The corresponding distance was calculated using the Haversine formula [19] as follows:

$$d=2r∙\arcsin\left(\sqrt{{sin}^{2}(\frac{\varphi_{2}-\varphi_{1}}{2})+cos\varphi_{1}∙cos\varphi_{2}∙{sin}^{2}(\frac{\lambda_{2}-\lambda_{1}}{2})}\right)$$

where d is the spherical distance, r is the radius of the sphere, φ is the latitude and λ is longitude of two points (1, 2).

Instead of using the closest measurement station to the center of each province, we conducted additional sensitivity analysis using the average daily maximum temperature from all measurement stations within the province (S1 Information).

### Mortality and population data

Death statistics from Statistics Korea for 1991–1995 and 2015–2019 were used for mortality data [20]. The all-cause mortality was used to assess the effect of temperature on mortality. Each value was aggregated as daily deaths by province, sex, and age group (-65: under 65 years; +65: 65 years or more). The population for whom the age at death was missing was excluded from the analysis. Population data from Statistics Korea by sex and age group in each province for the corresponding period were used [18].

### Statistical analysis

The unit of observation was the daily measurement. For the nonlinear association considering the time-lagged effect, distributed lag nonlinear models (DLNMs) were used to confirm the relationships between exposure, lag, and response [21]. In the first step, the DLNM was performed for each province, and in the second step, a multivariate meta-analysis of the cross-basis coefficients was performed to calculate the pooled coefficients. In each step, modelling was performed for all-cause deaths in the overall population as well as for males under 65, females under 65, males over 65, and females over 65. The model for each stratum outcome includes an interaction term between a period as an indicator and the cross-basis to construct a model for each period. Additionally, for each stratum outcome, we performed an analysis without the interaction term to calculate the overall effects across the two periods.

For the cross-basis, the relationships between daily maximum temperature and death were obtained from a quadratic B-spline with two internal knots at the 50^th^ and 90^th^ percentiles (4 degrees of freedom), and the relationships between lag and death, whose maximum lagged effect was 6 days, were obtained from a natural cubic spline with an intercept and two internal knots equally spaced on a logarithmic scale (4 degrees of freedom). The cross-basis parameters were referenced from a previous study [4], and sensitivity analyses were conducted based on variations in the parameters (S1 Information). The time trend was linear, a natural cubic spline of the day of the year with 4 degrees of freedom, an indicator of the day of the week, relative humidity as linear, and log-transformed population as offsets, were adjusted in the models as follows:

$$\log\left(\lambda \right)=\alpha +cb(T;\eta )+\sum {s(u}_{k};\beta )+log(p)$$

where *λ* is the expectation of daily death count with quasi-poisson distribution. *α* is intercept. *cb* is a cross-basis function, where *T* is daily maximum temperature with parameter *η*. *u_k_* are other covariates with *β* as parameters. p is popultion

Cross-basis coefficients were reduced to the overall cumulative relationship prior to meta-analysis [22]. The reduced model for each stratum outcome by each period was used to estimate the pooled effect across provinces through a meta-analysis. In the multivariate meta-analysis, the means and ranges of the daily maximum temperature by province were adjusted in the meta-regression to correct for heterogeneity in the exposure-response [4]. Pooled cross-basis coefficients were used to calculate the nonlinear cumulative relative risk (CRR) relationship, accounting for lagged effects. The KMA defines heat waves as days with a daily maximum temperature of 33°C or higher [14]. We used an absolute temperature threshold of 33°C as the reference temperature for estimating CRRs to compare the absolute effect sizes of high temperatures in the past and current periods and their changes over time. Differences in the nonlinear relationships by period, sex, and age group were tested using the multivariate Wald test for coefficients and their covariance matrices.

The attributable fraction (AF) and number were calculated by applying provincial, gender, and age-specific associations for each period, and the empirical confidence intervals (eCIs) of the attributable measures were calculated using Monte Carlo simulations with 1,000 samples of the coefficients [5] as follows:

$${AF}_{x,t}=1-exp(-\sum_{l=0}^{L}\beta_{x_{t},l})$$

$${AN}_{x,t}={AF}_{x,t}\sum_{l=0}^{L}\frac{n_{t+1}}{L+1}$$

where AF is an attributable fraction, AN is an attributable number. ∑*β* is the cumulative log-relative risk of temperature x_t_ on day t, n_t_ is the number of deaths on day t, and L is the maximum lag day.

To assess the impact of population aging on excess deaths at high temperatures, we hypothesized that changes in the age-gender distribution of the population over time could significantly influence mortality rates. We applied the gender-age distribution (proportion of the gender-age strata) from the past period (1991–1995) to the recent period’s observed person-years (2015–2019). This allowed us to estimate a “non-aging” scenario wherein we could observe excess deaths as if the age-gender structure of the population remained constant. We then applied the gender-age specific excess death rates, as modeled in 2015–2019, to this non-aging population structure. For 2018, we utilized the sex–age distribution from 1994, creating a direct, year-to-year comparative framework. This method enabled us to calculate the AFs and excess death rates under the non-aging scenario, thereby providing a clearer picture of standardized excess mortality than non-standardized figures.

### Ethics statement

This study used the death statistics data from Statistics Korea, which is a publicly available secondary data, and the data does not contain personal information. In addition, the data were completely anonymized, making it impossible to identify specific individuals. The research was a daily time-series design, and the data were processed and used as the number of deaths per day rather than at the individual level. Therefore, through exemption from IRB review, the need for consent was waived, and consent forms from subjects were not separately obtained. This study was approved by the Institutional Review Board of Seoul National University as a study using retrospective mortality data (No. E2110/002-005).

## Results

The mean (min, max) daily maximum temperature for summer was 28.0°C (17.7, 37.7) in 1991–1995 and 28.8°C (17.9, 38.3) in 2015–2019. The annual frequency of heat waves (days with a maximum temperature of 33°C or more) was 11.8 days in 1991–1995 and 18.8 days in 2015–2019. The number of deaths from all causes during the summer was 375,810 in 1991–1995 and 440,553 in 2015–2019, with mortality rate of 520.9 (/100,000 person-years) in 1991–1995 and 529.1 in 2015–2019 (Table 1).

**Table 1:** Distribution of weather and death descriptions in the past and in recent times of summer.

	Across cities		1991–1995	2015–2019
**Daily average temperature** (°C)	Mean (SD)		23.1 (3.4)	24.2 (3.3)
	Median (min., p25, p75, max.)		23.0 (14.2, 20.8, 25.5, 31.1)	24.0 (15.8, 21.7, 26.7, 32.6)
**Daily max. temperature** (°C)	Mean (SD)		28.0 (3.7)	28.8 (3.8)
	Median (min., p25, p75, max.)		28.0 (17.7, 25.4, 30.5, 37.7)	28.7 (17.9, 26.2, 31.4, 38.3)
**No. of heat wave days** (annual mean)			11.8	18.8
**Humidity** (%)	Mean (SD)		75.9 (9.7)	73.5 (13.5)
**Deaths**	**Total**	No. of deaths / person years	375,810 / 72,151,527	440,553 / 83,266,268
		Rates (/100,000PY)	520.9	529.1
	**Male, -65**	No. of deaths / person years	127,454 / 34,788,020	77,357 / 36,782,260
		Rates (/100,000PY)	366.4	210.3
	**Female, -65**	No. of deaths / person years	52,464 / 33,328,950	31,413 / 34,906,000
		Rates (/100,000PY)	157.4	90.0
	**Male, +65**	No. of deaths / person years	89,453 / 1,502,120	164,925 / 4,917,247
		Rates (/100,000PY)	5,955.1	3,354.0
	**Female, +65**	No. of deaths / person years	106,439 / 2,532,437	166,858 / 6,660,761
		Rates (/100,000PY)	4,203.0	2,505.1

SD: standard deviation, min: minimum, max: maximum, p25:25^th^ percentile, p75:75^th^ percentile, PY: person-years, -65: below 65 years old, +65: 65 or more years old. Each period was from June to September. Heat wave day defined as a day with a maximum daily temperature of 33°C or above.

**Fig 2:**
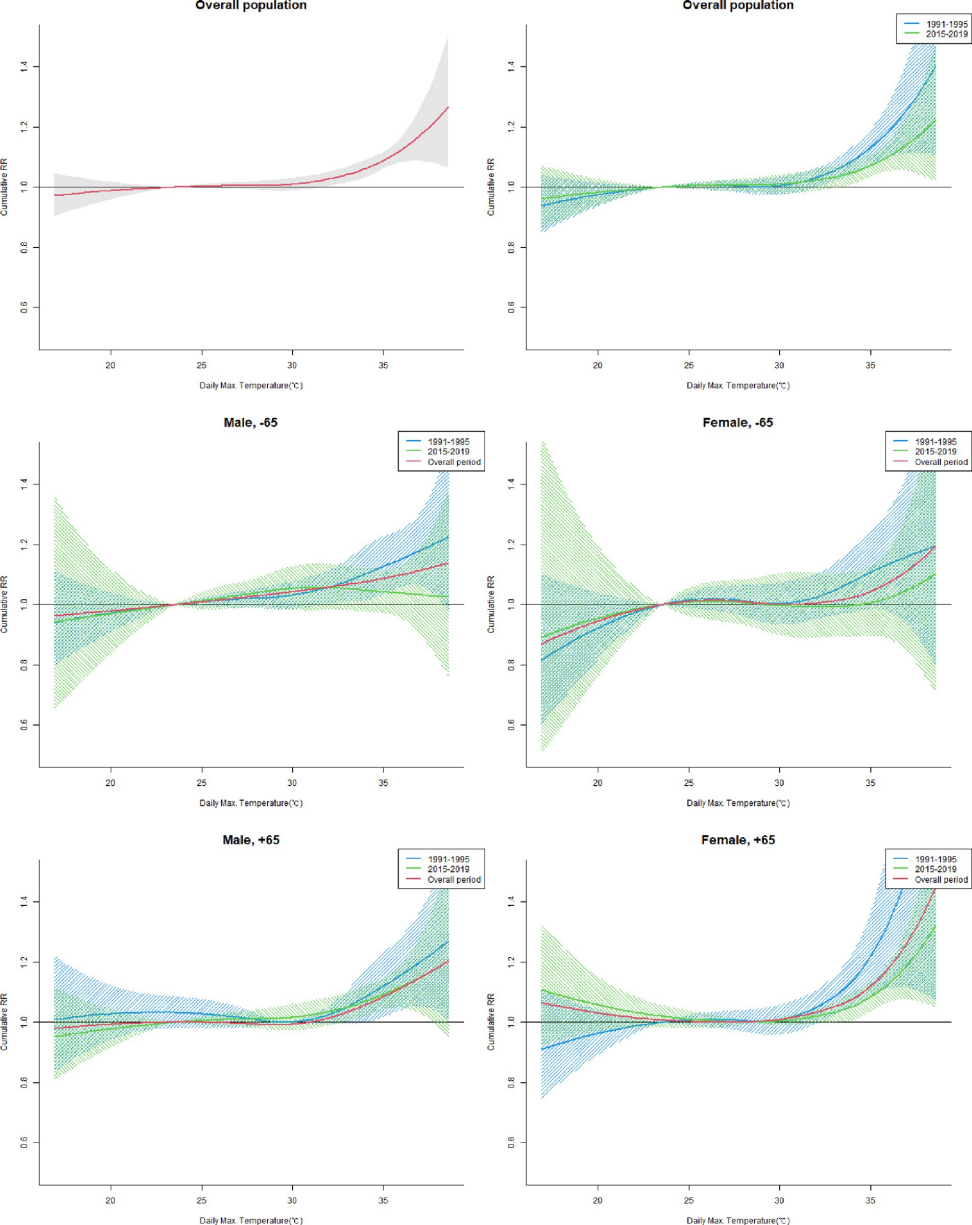
Nonlinear association between daily maximum temperature and mortality by period, gender and age group. Max.: maximum, -65: below 65 years old, +65: 65 or more years old. Each period covers June to September per year. The relationships between summer daily maximum temperature and mortality were determined through meta-analysis of coefficients estimated by distributed lag nonlinear models by province. The CRR in the plots is centered on the MMT (If the MMT is less than the 10th percentile, the 10th percentile was used as centering value). The red line represents the relationship over the entire period. Blue lines represent relationships in the period 1991–1995, and Green lines represent relationships in the period 2015–2019. The shaded area around the line means the 95% confidence interval.

S1 Table and Fig 2 show the nonlinear relationship between the daily maximum temperature and mortality. CRRs tended to be lower in the recent 5 years than in the past, higher in females than in males, and higher in those over 65 than in those under 65; however, the differences were not statistically significant (S1 Table, Fig 2; *P* = 0.428 for period differences, 0.511 for sex differences, and 0.063 for age differences). We performed a sensitivity analysis of the difference between the two periods according to the maximum lagged effect parameter change, and the results are presented it in S1 Information. The results of testing the differences between the two periods for each subpopulation (Male, under 65; Female, under 65; Male, over 65; Female, over 65) are presented in S2 Table. The nonlinear association between daily maximum temperature and mortality by year from 1991 to 2019 is depicted in S1 Fig. A gradual decline in the effect of daily maximum temperature on mortality was observed from 1991 to 2019.

In the summer of 1991–1995, the excess death rate was 4.2 per 100,000 person-years (3,012 / 72,151,527 person-years). Conversely, during the summers of 2015–2019, the excess death rate was 2.7 per 100,000 person-years (2,280 / 83,266,268). On the other hand, the excess death rate in the non-aging scenario was estimated at 1.2 per 100,000 person-years (993 / 83,266,268) in 2015–2019 (Table 2).

**Table 2:** Impacts of high air temperature of summer in Korea.

	Past		Recent (Aging case)		Non-aging scenario	
	1991–1995	1994	2015–2019	2018	2015–2019	2018
Person-year	72,151,527	14,566,888	83,266,268	16,716,190	83,266,268	16,716,190
Observed death counts	375,810	78,884	440,553	91,249	250403	51,123
Excess death counts (95% eCIs)	3,012 (1,591, 3,511)	2,567 (1,250, 3,055)	2,280 (445, 3,559)	1,392 (387, 1,994)	993	593
AF (95% eCIs) (%)	0.8 (0.4, 0.9)	3.3 (1.6, 3.9)	0.5 (0.1, 0.8)	1.5 (0.4, 2.2)	0.4	1.2
Excess death rate (95% eCIs) (/100,000PY)	4.2 (2.2, 4.9)	17.6 (8.6, 21.0)	2.7 (0.5, 4.3)	8.3 (2.3, 11.9)	1.2	3.6

AF: Attributable fraction, eCIs: empirical confidence Intervals, PY: person-years. Each period covers June to September per year

The non-aging scenario assumes that the gender-age distribution of person-years in recent periods was the same as that in past periods and applied the stratum-specific excess death rate. Observed death counts in the non-aging scenario were calculated by assuming the past gender-age distribution in the recent person-years.

Specifically, in the summer of 1994, the excess death rate was 17.6 per 100,000 person-years (2,567 / 14,566,888). In the summer of 2018, the excess death rate was 8.3 per 100,000 person-years (1,392 / 16,716,190) and that in the non-aging scenario was 3.6 per 100,000 person-years (593 / 16,716,190).

The AFs were lower at 0.8% for the period 1991–1995 and 0.5% for 2015–2019. In the non-aging scenario, the AFs in 2015–2019 dropped further to 0.4%, which was even lower than that in the recent aging case. Specifically, AFs were 3.3% in 1994 and 1.5% in 2018. Under the non-aging scenario in 2018, the AFs were estimated at 1.2%.

**Fig 3:**
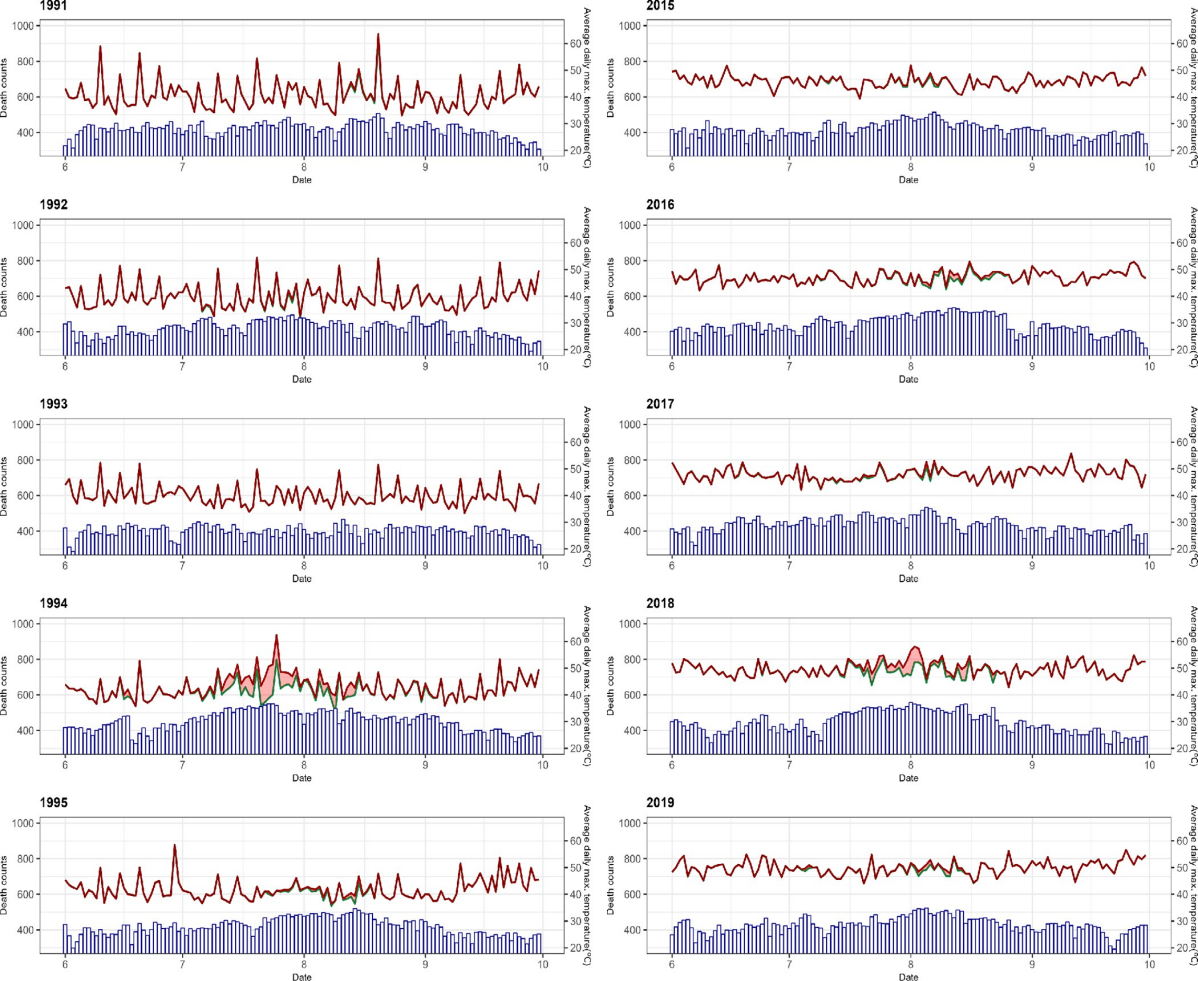
Average daily maximum temperature for summer and daily excess death count by year for the past and recent 5 years. The blue bar indicates the average daily maximum temperature. The red line represents the number of observed daily deaths, and the green line represents the number of deaths not attributable to high ambient temperature. The red area between the red and green lines indicates the daily excess death count attributed to high ambient temperature.

As illustrated in Fig 3, deaths attributable to high air temperatures occurred mainly in 1994 and 2018 during the worst heat waves, and excess deaths were lower in the most recent period than in the past.

The provincial characteristics of location, population, daily maximum temperature, and impact of high air temperature by province are described in S3 and S4 Tables.

## Discussion

In this study, the association between high temperatures and mortality was confirmed by estimating the Korean integrated exposure-response curve by evaluating each province. The effects of high temperatures on mortality affected the vulnerable population over 65 compared to those under 65, although the difference was borderline significant. It has also been suggested that women are more susceptible than men to high temperatures. When comparing the two periods, the point estimates for the effects of high temperatures on mortality were lower in the recent period compared to the past, although the difference was not statistically significant. Notably, among women aged 65 and over, the difference in the nonlinear relationship between the two periods was borderline significant, with sensitivity analysis showing a statistically significant difference (S1 Information). Recently, the number of excess deaths due to high temperatures has decreased.

The association between high temperature exposure and mortality has been consistently reported in previous studies. In Brisbane, Melbourne, and Sydney, relative risks (RRs) for non-external deaths on heatwave days (defined as days with daily average temperatures above the 95th percentile) compared to non-heatwave days were 1.13 (95% CIs 1.08–1.19), 1.10 (1.06–1.14), and 1.06 (1.01–1.10), respectively [10]. According to a cross-national study, there is an association between high air temperature and total mortality in all countries, with the highest RRs in Spain and Australia and an RR of 1.015 in Korea when comparing the 90th percentile of daily average temperatures to the minimum mortality percentile [4]. In a study that examined 400 cities in 18 countries, a significant association was found with heat waves [6]. Although statistical significance of CRR differences by sex and age was not reached in this study, and the differences were small, it is consistent with the findings of greater RR in the elderly and women in the analysis of previous studies [7–10, 23, 24]. Our sensitivity analysis indicated that the statistically significant higher relative risks observed in the elderly and elderly women can support these findings (S1 Information). The ability of the elderly to cope with high temperatures may decrease because of underlying diseases and deterioration of their physical function [25]. In women, the difference in effects can be explained by the dysfunction of the thermoregulation, the deterioration of cardiovascular health due to the side effects of menopause [26], and the difference in socioeconomic status between the sexes [27].

In this study, the point estimates of RR were calculated to be small in recent periods compared with past periods. The results of previous studies are not completely consistent with our results, with AF decreasing recently and some RR increasing compared to the past in Korea [28]. These discrepancies may stem from various factors, such as differences in the study period, differences in weather stations, absence of age-specific analysis, and divergence in detailed model settings. Acknowledging these potential sources of variation is crucial to understand the complexities of heat wave impact and refining future research methodologies. When the maximum-lagged effect was 3 days, the difference in effect between the two periods was statistically significant (S1 Information), suggesting that there is a difference in the effect of short-term exposure. Although we did not observe a significant difference between the two periods in this model, owing to the small decrease in the effect and lack of statistical power, the observation of this trend may have implications. It is assumed that socioeconomic levels have improved compared to that in the past. As science and technology have advanced, the impacts may have decreased owing to environmental improvements, such as improvements in the quality of medical care, increased distribution of air conditioning at home and at work (according to Statistics Korea [18], the penetration rate of air conditioning increased from 0.09 per household in 1994 to 0.78 per household in 2013), and improvements in community infrastructure. These findings suggest the potential value of implementing heat prevention measures and their possible effectiveness. In addition, population aging has played a significant role in the changes observed in the number of excess deaths from past to recent periods. Assuming a non-aging scenario, the number of excess deaths was even reduced by approximately 56.5%, from 2,280 in the recent aging case to 993 in the non-aging scenario. This substantial reduction underscores the significant impact of aging populations on the burden of high temperatures. As aging is likely to pose greater challenges in the future, it is crucial to incorporate this factor into adaptation policies. Proactive and well-tailored responses are essential to mitigate the heightened risks and vulnerabilities associated with an aging demographic and ensure more effective management of heat-related health impacts.

In our analysis of the impacts of high air temperatures across provinces (S4 Table), although we observed a consistent trend of decreasing AF in recent periods compared to the past, the AF varied heterogeneously across provinces. This variability may be influenced by the presence of elderly populations or economically vulnerable groups in specific areas [29], suggesting that these heterogeneous results could reflect regional characteristics such as socioeconomic status, proportion of elderly population, and infrastructure resilience. Additionally, urban areas or regions lacking green spaces may experience more pronounced effects due to the urban heat island effect [30]. Air pollution may also interact with heatwaves [31], playing a role in modulating the AF in areas with high pollution levels. Future research should focus on identifying modifiers that contribute to these regional differences.

South Korea established the 2^nd^ Basic Plan for Climate Change Response in October 2019, which aimed to reduce greenhouse gas emissions to 536 million tons by 2030, prepare for abnormal climate conditions, and strengthen all sectors to implement the Paris Agreement [32]. This plan includes not only plans to reduce greenhouse gas emissions but also plans to establish a foundation for minimizing health damage (prioritizing health adaptation policies, strengthening monitoring, and establishing the basis for a national health impact assessment), strengthening the protection of the vulnerable (developing tailored support projects and introducing health management and risk prevention programs for high-risk groups), and strengthening the management and system of cooperation (organization in charge of the health adaptation project and operation of a medical emergency room). To prepare for future climate change, it is necessary to take more efficient preventive measures to prepare for high summer temperatures (*e*.*g*., more thermal shelters, expansion of local infrastructure, informing the public about risks, and providing education) and to continuously share and use health-related databases. To achieve this objective, it is essential to establish systems that monitor the exposure to high temperatures and their health effects, thus requiring collaboration with key institutions, including the Korea Disease Control and Prevention Agency, the Ministry of Environment, and other relevant organizations such as the National Health Insurance Corporation and Statistics Korea. As the emergency room surveillance system for heat-related illness in Korea is a sample surveillance system, it does not cover all patients with heat illness. Therefore, policies are needed to increase the number of monitoring agencies. It may also be necessary to provide an application or platform through which the public can identify the risk of high temperatures in real time.

The strength of the method used in this study is that it examined the entire country spatially and at the two hottest periods temporally. Earlier Korean studies had limitations in that they did not cover the entire country, and there were few temporal comparisons. Previous Korean studies focused on Seoul [29, 33–36] or only large cities [37–42], and integrated relationships were not calculated. Some studies modeled the entire study period but did not present analyses of differences by period [34–39, 42, 43]. In this study, two time periods with long-term intervals, including the worst heat waves, were compared. The inclusion of these periods ensured that our data had a large temperature variation and that the effects of high air temperatures could be well demonstrated between them.

This study also has the strengths of modeling associations and evaluating excess deaths. In terms of modeling associations, most previous Korean studies presented a dichotomous or linear relationship per unit increase above a threshold as the main outcome and did not consider exposures with a distributed lag. One study [42] performed modeling by defining exposure to high temperatures as a dichotomous type of heat or non-heat wave. Some studies did not describe nonlinear relationships between continuous temperature exposure and mortality or applied a natural spline to temperature exposure in generalized linear models or generalized additive models only to visualize the nonlinear relationship and presented an estimate as a linear association above the threshold using a truncated linear function or piecewise linear regression [29, 33, 34, 37–41, 43]. In addition, some studies have modelled without considering the lag effect [42] and others have included analysis using either individual single lag or moving average [29, 33, 37, 39, 41, 43]. Most previous Korean studies did not calculate excess deaths [29, 33, 35–43], and one of the studies [34] was limited in that it was estimated only in Seoul using a linear association on the day. In this study, after the DLNM analysis considering the time-lag effect in the province, the Korean integrated nonlinear CRR relationship was estimated using a meta-analysis of the cross-basis coefficient, and excess deaths were estimated. The strength of this study is that a more detailed assessment of exposure was made, including an overall map of Korea, and a more detailed nonlinear relationship was estimated considering regional heterogeneity.

However, because Sejong and Ulsan were excluded from the study, as well as deaths of unknown age, there could be a potential for selection bias. Because of the constraints of the research design rather than individual-level data, there is a risk of information bias, in that temperature exposure by province may differ from an individual’s actual temperature exposure. Additionally, using temperature data from the station nearest the central coordinates of the region might introduce a measurement error.

In this study, a fixed threshold of 33°C was adopted, contrasting with the frequent use of relative metrics such as Minimum Mortality Temperature (MMT) or percentiles in previous studies. Using relative values as thresholds has the advantage of allowing for relative comparisons across various periods and populations. Conversely, the application of an absolute threshold enables consistent and direct comparisons within its defined range, thereby offering a simpler interpretation. It is important to note that when evaluating differences in RRs across regions or periods, careful consideration is needed regarding whether to use relative thresholds or absolute thresholds, as this choice can influence the interpretation of whether the estimated effect size reflects the population’s vulnerability or adaptation. Future studies should focus on these aspects when determining an appropriate threshold. Moreover, additional studies are required to ascertain the most suitable threshold choice based on specific research objectives. In addition, the effects of high temperatures may interact with air pollutants [44], so improved modelling is needed to account for these. The scope should also be expanded to various specific diseases such as kidney disease, including not only mortality but also morbidity, and vulnerable groups such as those with underlying diseases, the disabled, pregnant women, and occupations should be analyzed in future studies.

## Conclusion

The impact of high air temperatures might have diminished recently as compared to that in the past possibly due to adaptations to changes in demographic, social, and environmental factors across these distinct periods. Notably, aging significantly influenced the mortality burden of high air temperatures. Because aging will remain a concern in the future, policies should address these vulnerabilities. Further research is necessary to uncover factors related to societal resilience to climate change.

## Supporting information

S1 Table CRR on mortality of high daily maximum temperature for summer by period, gender and age group. (DOCX)

S2 Table Tests for the difference between 1991–1995 and 2015–2019. (DOCX)

S3 Table Provincial characteristics on location, population, and daily maximum temperature. (DOCX)

S4 Table Impacts of high air temperature of summer by provinces. (DOCX)

S1 Fig Nonlinear association between daily maximum temperature and mortality by year from 1991 to 2019. (DOCX)

S1 Information Sensitivity analysis for maximum-lagged effect, other cross-basis parameters, and temperature assessment. (DOCX)
