# Supplementary material for: Excess mortality related to high air temperature: Comparison of the periods including 1994 and 2018, the worst heat waves in the history of South Korea
Source: PLoS One. 2024 Nov 13;19(11):e0310797. doi: 10.1371/journal.pone.0310797 (PMC11560060; doi:10.1371/journal.pone.0310797)
Supplement: S1 Table — (DOCX) [file pone.0310797.s001.docx]

**S1 Table. CRR on mortality of high daily maximum temperature for summer by period, gender and age group**

|  | **Study periods** | | **Gender** | | **Age** | |
| --- | --- | --- | --- | --- | --- | --- |
|  | **1991–1995** | **2015–2019** | **Male** | **Female** | **-65** | **+65** |
| **Daily maximum temperatures** | CRR (95% CI) | CRR (95% CI) | CRR (95% CI) | CRR (95% CI) | CRR (95% CI) | CRR (95% CI) |
| 33.3℃ | 1.01 (1.00, 1.02) | 1.00 (1.00, 1.01) | 1.01 (1.00, 1.01) | 1.01 (1.00, 1.01) | 1.00 (1.00, 1.01) | 1.01 (1.00, 1.01) |
| 33.6℃ | 1.02 (1.01, 1.03) | 1.01 (1.00, 1.01) | 1.01 (1.00, 1.02) | 1.01 (1.00, 1.02) | 1.01 (1.00, 1.01) | 1.01 (1.01, 1.02) |
| 33.8℃ | 1.02 (1.01, 1.04) | 1.01 (1.00, 1.02) | 1.01 (1.00, 1.02) | 1.02 (1.01, 1.03) | 1.01 (1.00, 1.02) | 1.02 (1.01, 1.03) |
| 34.1℃ | 1.03 (1.02, 1.05) | 1.02 (1.01, 1.03) | 1.02 (1.01, 1.03) | 1.02 (1.01, 1.04) | 1.01 (1.00, 1.02) | 1.03 (1.02, 1.04) |
| 34.3℃ | 1.04 (1.02, 1.06) | 1.02 (1.01, 1.03) | 1.02 (1.01, 1.03) | 1.03 (1.02, 1.04) | 1.01 (1.00, 1.03) | 1.03 (1.02, 1.04) |
| 34.6℃ | 1.06 (1.03, 1.08) | 1.03 (1.01, 1.04) | 1.03 (1.01, 1.04) | 1.04 (1.03, 1.06) | 1.02 (1.00, 1.04) | 1.04 (1.03, 1.06) |
| 34.9℃ | 1.07 (1.04, 1.10) | 1.03 (1.02, 1.05) | 1.03 (1.02, 1.05) | 1.05 (1.03, 1.08) | 1.02 (1.00, 1.04) | 1.05 (1.04, 1.07) |
| 35.3℃ | 1.09 (1.04, 1.13) | 1.04 (1.02, 1.07) | 1.04 (1.02, 1.06) | 1.07 (1.04, 1.10) | 1.03 (1.00, 1.05) | 1.07 (1.04, 1.10) |
| 35.8℃ | 1.11 (1.05, 1.18) | 1.06 (1.02, 1.10) | 1.05 (1.02, 1.09) | 1.10 (1.05, 1.14) | 1.04 (1.00, 1.07) | 1.09 (1.05, 1.14) |
| 36.6℃ | 1.16 (1.06, 1.28) | 1.09 (1.01, 1.17) | 1.07 (1.01, 1.14) | 1.15 (1.07, 1.24) | 1.05 (0.99, 1.12) | 1.13 (1.05, 1.22) |
| 38.0℃ | 1.28 (1.06, 1.54) | 1.15 (0.99, 1.35) | 1.11 (0.97, 1.27) | 1.28 (1.09, 1.50) | 1.09 (0.95, 1.25) | 1.23 (1.04, 1.44) |
| *P* value | 0.428 | | 0.511 | | 0.063 | |

-65: below 65 years old, +65: 65 or more years old, CRR: cumulative relative risk, CI: confidence interval. CRRs were calculated through meta-analysis of coefficients estimated by distributed lag nonlinear models according to the province. CRRs are relative values that compare the mortality risks at 33℃. Each period covers June to September per year. Daily maximum temperatures are the average of 90th, 91th, 92th, 93th, 94th, 95th, 96th, 97th, 98th, 99th, and 99.9th percentile for each region across the two periods.
