# Supplementary material for: Excess mortality related to high air temperature: Comparison of the periods including 1994 and 2018, the worst heat waves in the history of South Korea
Source: PLoS One. 2024 Nov 13;19(11):e0310797. doi: 10.1371/journal.pone.0310797 (PMC11560060; doi:10.1371/journal.pone.0310797)
Supplement: S2 Table — (DOCX) [file pone.0310797.s002.docx]

**S2 Table. Tests for the difference between 1991-1995 and 2015-2019**

|  | Nonlinear relationship (cross-basis coefficients) | CRR at 99th percentile |
| --- | --- | --- |
|  | P-value for the difference between 1991-1995 and 2015-2019 | |
| Overall population | 0.428 | 0.259 |
| Male, -65 | 0.280 | 0.159 |
| Female, -65 | 0.833 | 0.668 |
| Male, +65 | 0.678 | 0.536 |
| Female, +65 | 0.080 | 0.197 |

-65: below 65 years old, +65: 65 or more years old. Differences in the nonlinear relationships were tested using the multivariate Wald test. Differences in CRR at 99th percentile, centered at 33℃, were tested using Z-test
