# Supplementary material for: Excess mortality related to high air temperature: Comparison of the periods including 1994 and 2018, the worst heat waves in the history of South Korea
Source: PLoS One. 2024 Nov 13;19(11):e0310797. doi: 10.1371/journal.pone.0310797 (PMC11560060; doi:10.1371/journal.pone.0310797)
Supplement: S3 Table — (DOCX) [file pone.0310797.s003.docx]

**S3 Table. Provincial characteristics on Location, Population, and Daily Maximum Temperature**

|  |  |  | **Average population** | | **Daily maximum temperature (℃)** | | | | | | |
| --- | --- | --- | --- | --- | --- | --- | --- | --- | --- | --- | --- |
|  |  |  |  |  | Average | | Minimum | | Maximum | |  |
| **Provinces** | **Latitude** | **Longitude** | 1991-1995 | 2015-2019 | 1991-1995 | 2015-2019 | 1991-1995 | 2015-2019 | 1991-1995 | 2015-2019 |  |
| Seoul | 37.57 | 126.98 | 10,410,926 | 9,783,330 | 27.7 | 29.5 | 17.7 | 19.2 | 38.4 | 39.6 |  |
| Busan | 35.18 | 129.08 | 3,836,769 | 3,419,284 | 26.7 | 27.6 | 18.2 | 19.1 | 35.8 | 37.3 |  |
| Daegu | 35.87 | 128.60 | 2,405,674 | 2,454,005 | 29.3 | 28.3 | 18.9 | 13.9 | 39.4 | 39.2 |  |
| Incheon | 37.46 | 126.71 | 2,154,849 | 2,919,392 | 26.6 | 27.6 | 18.1 | 19.2 | 37.2 | 36.0 |  |
| Gwngju | 35.16 | 126.85 | 1,214,257 | 1,498,082 | 28.6 | 29.7 | 18.7 | 19.5 | 38.5 | 38.5 |  |
| Daejeon | 36.35 | 127.38 | 1,184,302 | 1,526,600 | 28.6 | 29.5 | 18.1 | 17.4 | 37.7 | 39.4 |  |
| Gyeonggi-do | 37.41 | 127.52 | 7,002,132 | 12,815,655 | 28.0 | 29.5 | 16.7 | 18.9 | 37.6 | 40.1 |  |
| Gangwon-do | 37.82 | 128.16 | 1,514,875 | 1,519,129 | 26.7 | 27.8 | 14.1 | 17.8 | 36.3 | 37.7 |  |
| Chungcheongbuk-do | 36.80 | 127.70 | 1,398,515 | 1,608,743 | 28.7 | 29.7 | 17.8 | 17.6 | 37.8 | 39.1 |  |
| Chungcheongnam-do | 36.52 | 126.80 | 1,866,589 | 2,149,956 | 28.3 | 27.4 | 18.4 | 11.7 | 37.7 | 37.0 |  |
| Jeollabuk-do | 35.72 | 127.15 | 1,972,938 | 1,824,471 | 28.8 | 29.4 | 18.2 | 19.6 | 38.2 | 38.9 |  |
| Jeollanam-do | 34.87 | 126.99 | 2,239,516 | 1,790,617 | 27.9 | 28.6 | 18.5 | 20.1 | 38.7 | 36.8 |  |
| Gyeongsangbuk-do | 36.49 | 128.89 | 2,719,502 | 2,675,042 | 28.1 | 28.4 | 16.3 | 16.2 | 36.8 | 38.1 |  |
| Gyeonsangnam-do | 35.46 | 128.21 | 2,859,216 | 3,342,549 | 28.7 | 30.0 | 18.4 | 18.5 | 39.2 | 39.5 |  |
| Jeju-do | 33.49 | 126.50 | 510,859 | 632,904 | 27.1 | 28.3 | 18.1 | 20.1 | 35.8 | 37.0 |  |
