# Supplementary material for: Excess mortality related to high air temperature: Comparison of the periods including 1994 and 2018, the worst heat waves in the history of South Korea
Source: PLoS One. 2024 Nov 13;19(11):e0310797. doi: 10.1371/journal.pone.0310797 (PMC11560060; doi:10.1371/journal.pone.0310797)
Supplement: S4 Table — (DOCX) [file pone.0310797.s004.docx]

**S4 Table. Impacts of high air temperature of summer by provinces**

|  | Person-years | | Observed Death | | Excess death counts (95% eCIs) | | AF (95% eCIs) (%) | | Excess death rate (95% eCIs) (/100,000PY) | |
| --- | --- | --- | --- | --- | --- | --- | --- | --- | --- | --- |
|  | 1991-1995 | 2015-2019 | 1991-1995 | 2015-2019 | 1991-1995 | 2015-2019 | 1991-1995 | 2015-2019 | 1991-1995 | 2015-2019 |
| Seoul | 433,659 | 1,206,996 | 17,797 | 24,492 | 722 (462, 936) | 582 (270, 852) | 4.1 (2.6, 5.3) | 2.4 (1.1, 3.5) | 166.4 (106.6, 215.8) | 48.2 (22.3, 70.6) |
| Busan | 169,551 | 514,994 | 7,067 | 12,401 | 23 (-124, 90) | 20 (-245, 212) | 0.3 (-1.8, 1.3) | 0.2 (-2.0, 1.7) | 13.5 (-73.0, 53.3) | 3.9 (-47.6, 41.2) |
| Daegu | 111,389 | 326,342 | 4,523 | 7,997 | 235 (-113, 462) | 244 (135, 315) | 5.2 (-2.5, 10.2) | 3.1 (1.7, 3.9) | 211.2 (-101.3, 414.7) | 74.8 (41.4, 96.6) |
| Incheon | 95,486 | 318,935 | 3,494 | 8,315 | 55 (-42, 101) | 102 (-266, 343) | 1.6 (-1.2, 2.9) | 1.2 (-3.2, 4.1) | 57.7 (-43.5, 105.7) | 32.1 (-83.5, 107.4) |
| Gwngju | 58,759 | 172,048 | 2,589 | 4,662 | 35 (-90, 112) | 45 (-4, 69) | 1.3 (-3.5, 4.3) | 1.0 (-0.1, 1.5) | 58.9 (-153.3, 191.2) | 26.2 (-2.2, 40.3) |
| Daejeon | 54,134 | 167,787 | 2,207 | 4,223 | 87 (32, 116) | 40 (13, 55) | 3.9 (1.4, 5.3) | 0.9 (0.3, 1.3) | 160.7 (58.5, 214.3) | 23.7 (8.0, 32.9) |
| Gyeonggi-do | 331,692 | 1,354,789 | 13,634 | 32,828 | 635 (402, 815) | 533 (23, 947) | 4.7 (3.0, 6.0) | 1.6 (0.1, 2.9) | 191.4 (121.3, 245.6) | 39.3 (1.7, 69.9) |
| Gangwon-do | 112,335 | 259,439 | 4,671 | 6,937 | 32 (-87, 90) | 14 (-146, 134) | 0.7 (-1.9, 1.9) | 0.2 (-2.1, 1.9) | 28.6 (-77.8, 80.5) | 5.4 (-56.3, 51.8) |
| Chungcheongbuk-do | 108,744 | 236,844 | 4,463 | 6,610 | 183 (1, 297) | 53 (-95, 193) | 4.1 (0.0, 6.7) | 0.8 (-1.4, 2.9) | 168.2 (0.5, 273.5) | 22.5 (-40.2, 81.4) |
| Chungcheongnam-do | 168,794 | 337,922 | 6,782 | 9,272 | 57 (-143, 152) | 73 (-125, 199) | 0.8 (-2.1, 2.2) | 0.8 (-1.4, 2.2) | 34.0 (-84.6, 89.9) | 21.6 (-37.1, 59.0) |
| Jeollabuk-do | 169,456 | 331,737 | 6,903 | 9,212 | 167 (-27, 285) | 61 (-67, 156) | 2.4 (-0.4, 4.1) | 0.7 (-0.7, 1.7) | 98.5 (-16.2, 167.9) | 18.5 (-20.1, 47.0) |
| Jeollanam-do | 219,173 | 380,423 | 9,793 | 10,896 | 110 (-31, 201) | 46 (-116, 146) | 1.1 (-0.3, 2.0) | 0.4 (-1.1, 1.3) | 50.2 (-14.3, 91.5) | 12.1 (-30.6, 38.3) |
| Gyeongsangbuk-do | 248,668 | 485,726 | 10,462 | 13,350 | 348 (171, 437) | 205 (130, 252) | 3.3 (1.6, 4.2) | 1.5 (1.0, 1.9) | 139.9 (68.6, 175.6) | 42.2 (26.7, 51.9) |
| Gyeonsangnam-do | 211,031 | 479,340 | 10,559 | 13,433 | 338 (54, 548) | 240 (-70, 503) | 3.2 (0.5, 5.2) | 1.8 (-0.5, 3.7) | 160.3 (25.7, 259.5) | 50.1 (-14.7, 105.0) |
| Jeju-do | 39,567 | 87,441 | 1,495 | 2,230 | -15 (-88, 18) | 21 (-2, 36) | -1.0 (-5.9, 1.2) | 1.0 (-0.1, 1.6) | -37.6 (-221.8, 45.4) | 24.4 (-2.2, 41.0) |
