# Supplementary material for: Excess mortality related to high air temperature: Comparison of the periods including 1994 and 2018, the worst heat waves in the history of South Korea
Source: PLoS One. 2024 Nov 13;19(11):e0310797. doi: 10.1371/journal.pone.0310797 (PMC11560060; doi:10.1371/journal.pone.0310797)
Supplement: S1 Fig — (DOCX) [file pone.0310797.s005.docx]

**S1 Figure. Nonlinear association between daily maximum temperature and mortality by year from 1991 to 2019.**


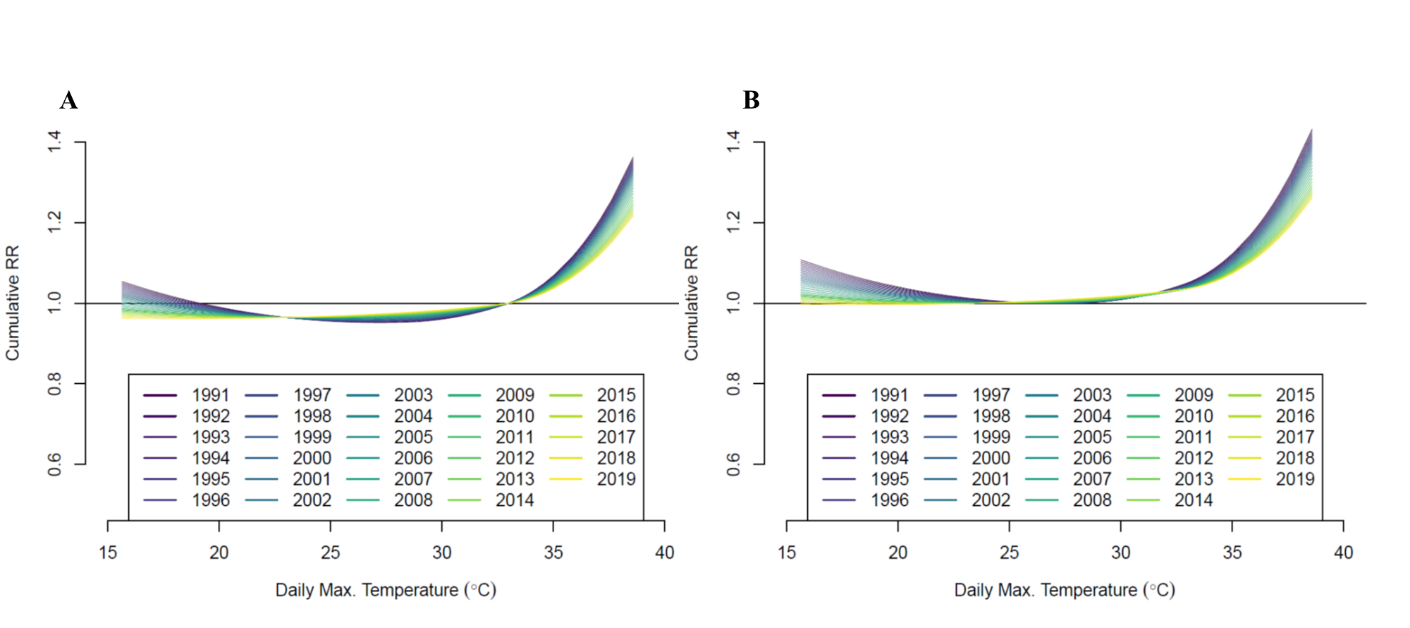


Modeling and meta-analysis were conducted by incorporating a linear interaction term of cross-basis and year into the model. 'A' represents the association using 33℃ as the threshold, while 'B' depicts the association using MMT as the threshold.
