# Supplementary material for: Excess mortality related to high air temperature: Comparison of the periods including 1994 and 2018, the worst heat waves in the history of South Korea
Source: PLoS One. 2024 Nov 13;19(11):e0310797. doi: 10.1371/journal.pone.0310797 (PMC11560060; doi:10.1371/journal.pone.0310797)
Supplement: S1 Information — (DOCX) [file pone.0310797.s006.docx]

**S1 Information. Sensitivity analysis for maximum-lagged effect, other cross-basis parameters, and temperature assessment.**

**1. Sensitivity analysis for maximum-lagged effect**

In the overall population, the lag-response plot at the 99th percentile indicated that the mortality effect was negligible beyond 6 days. Therefore, we determined that setting the maximum lagged effect to 6 days would be sufficient and proceeded with modeling accordingly.


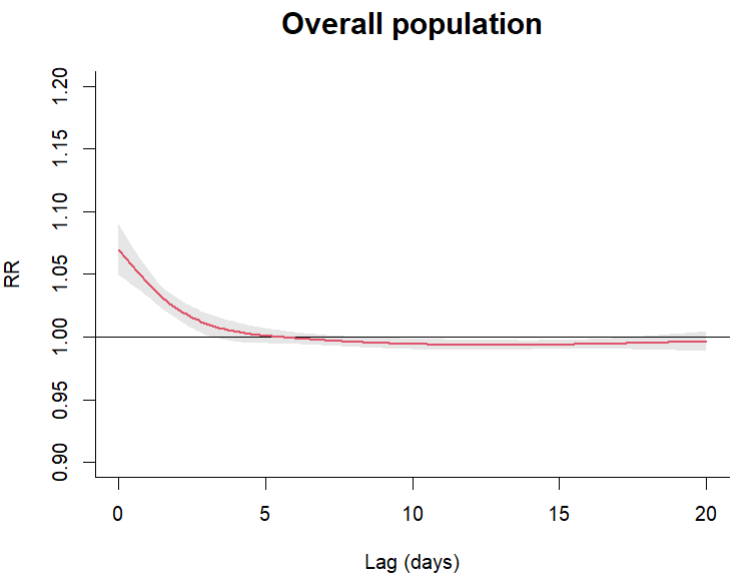


In addition, we performed sensitivity analyses with different maximum lagged effect to ensure robustness.

**CRR on Mortality of High Daily Maximum Temperature for Summer by Period, Gender, and Age Group According to Maximum Lagged Effect**

| Maximum-lagged effect | Daily maximum temperatures | Study periods | | Gender | | Age | |
| --- | --- | --- | --- | --- | --- | --- | --- |
|  |  | 1991–1995 | 2015–2019 | Male | Female | -65 | +65 |
|  |  | CRR (95% CI) | | | | | |
| Lag 3 | 95th | 1.04 (1.03, 1.06) | 1.02 (1.01, 1.03) | 1.03 (1.02, 1.04) | 1.03 (1.02, 1.04) | 1.02 (1.00, 1.03) | 1.04 (1.03, 1.05) |
|  | 96th | 1.06 (1.03, 1.08) | 1.03 (1.02, 1.04) | 1.03 (1.02, 1.05) | 1.04 (1.03, 1.06) | 1.02 (1.00, 1.04) | 1.05 (1.03, 1.06) |
|  | 97th | 1.07 (1.04, 1.10) | 1.04 (1.02, 1.05) | 1.04 (1.03, 1.06) | 1.06 (1.04, 1.08) | 1.03 (1.01, 1.04) | 1.06 (1.04, 1.08) |
|  | 98th | 1.09 (1.04, 1.14) | 1.05 (1.02, 1.07) | 1.05 (1.03, 1.07) | 1.08 (1.05, 1.11) | 1.03 (1.01, 1.06) | 1.08 (1.05, 1.11) |
|  | 99th | 1.13 (1.05, 1.22) | 1.07 (1.03, 1.12) | 1.06 (1.02, 1.11) | 1.13 (1.07, 1.18) | 1.04 (1.00, 1.09) | 1.11 (1.06, 1.17) |
|  | 99.9th | 1.22 (1.04, 1.43) | 1.12 (1.02, 1.23) | 1.09 (0.98, 1.20) | 1.24 (1.11, 1.38) | 1.07 (0.96, 1.18) | 1.20 (1.07, 1.34) |
|  | P value | 0.045 | | 0.101 | | 0.048 | |
| Lag 4 | 95th | 1.05 (1.03, 1.08) | 1.02 (1.01, 1.04) | 1.03 (1.02, 1.04) | 1.04 (1.02, 1.05) | 1.02 (1.00, 1.04) | 1.04 (1.02, 1.05) |
|  | 96th | 1.06 (1.03, 1.09) | 1.03 (1.01, 1.04) | 1.03 (1.02, 1.05) | 1.05 (1.03, 1.07) | 1.02 (1.01, 1.04) | 1.05 (1.03, 1.06) |
|  | 97th | 1.08 (1.04, 1.12) | 1.04 (1.02, 1.06) | 1.04 (1.02, 1.06) | 1.06 (1.04, 1.08) | 1.03 (1.01, 1.05) | 1.06 (1.04, 1.08) |
|  | 98th | 1.10 (1.04, 1.16) | 1.05 (1.02, 1.08) | 1.05 (1.02, 1.08) | 1.08 (1.05, 1.12) | 1.04 (1.01, 1.06) | 1.08 (1.04, 1.12) |
|  | 99th | 1.14 (1.04, 1.24) | 1.07 (1.02, 1.13) | 1.07 (1.02, 1.12) | 1.13 (1.06, 1.19) | 1.05 (1.00, 1.10) | 1.12 (1.06, 1.18) |
|  | 99.9th | 1.22 (1.03, 1.45) | 1.12 (1.01, 1.25) | 1.10 (0.99, 1.23) | 1.23 (1.09, 1.39) | 1.07 (0.96, 1.20) | 1.21 (1.07, 1.36) |
|  | P value | 0.170 | | 0.212 | | 0.193 | |
| Lag 5 | 95th | 1.05 (1.03, 1.08) | 1.03 (1.01, 1.04) | 1.03 (1.02, 1.04) | 1.04 (1.03, 1.06) | 1.02 (1.00, 1.04) | 1.04 (1.03, 1.06) |
|  | 96th | 1.07 (1.04, 1.10) | 1.03 (1.02, 1.05) | 1.04 (1.02, 1.05) | 1.05 (1.03, 1.07) | 1.02 (1.00, 1.04) | 1.06 (1.04, 1.07) |
|  | 97th | 1.08 (1.04, 1.13) | 1.04 (1.02, 1.06) | 1.04 (1.03, 1.06) | 1.07 (1.04, 1.10) | 1.03 (1.01, 1.05) | 1.07 (1.05, 1.09) |
|  | 98th | 1.11 (1.05, 1.17) | 1.06 (1.02, 1.09) | 1.06 (1.03, 1.09) | 1.09 (1.06, 1.13) | 1.04 (1.01, 1.07) | 1.09 (1.05, 1.13) |
|  | 99th | 1.16 (1.06, 1.27) | 1.08 (1.02, 1.15) | 1.08 (1.02, 1.14) | 1.14 (1.08, 1.22) | 1.05 (1.00, 1.11) | 1.13 (1.06, 1.21) |
|  | 99.9th | 1.27 (1.05, 1.53) | 1.14 (1.00, 1.30) | 1.12 (0.98, 1.28) | 1.26 (1.11, 1.44) | 1.08 (0.96, 1.22) | 1.23 (1.07, 1.42) |
|  | P value | 0.394 | | 0.383 | | 0.108 | |
| Lag 7 | 95th | 1.06 (1.03, 1.09) | 1.03 (1.01, 1.04) | 1.03 (1.01, 1.04) | 1.04 (1.02, 1.06) | 1.02 (1.00, 1.03) | 1.04 (1.03, 1.06) |
|  | 96th | 1.07 (1.03, 1.11) | 1.04 (1.02, 1.05) | 1.03 (1.01, 1.05) | 1.06 (1.03, 1.08) | 1.02 (1.00, 1.04) | 1.05 (1.03, 1.07) |
|  | 97th | 1.09 (1.04, 1.14) | 1.04 (1.02, 1.07) | 1.04 (1.02, 1.06) | 1.07 (1.04, 1.10) | 1.03 (1.00, 1.05) | 1.07 (1.04, 1.10) |
|  | 98th | 1.12 (1.05, 1.19) | 1.06 (1.02, 1.10) | 1.05 (1.01, 1.09) | 1.10 (1.06, 1.15) | 1.04 (1.00, 1.07) | 1.09 (1.05, 1.14) |
|  | 99th | 1.18 (1.07, 1.31) | 1.09 (1.01, 1.17) | 1.07 (1.00, 1.14) | 1.16 (1.08, 1.25) | 1.06 (1.00, 1.13) | 1.13 (1.05, 1.22) |
|  | 99.9th | 1.32 (1.07, 1.62) | 1.15 (0.98, 1.35) | 1.10 (0.95, 1.29) | 1.32 (1.12, 1.54) | 1.12 (0.97, 1.28) | 1.23 (1.04, 1.46) |
|  | P value | 0.290 | | 0.255 | | 0.075 | |
| Lag 8 | 95th | 1.06 (1.03, 1.08) | 1.03 (1.01, 1.04) | 1.03 (1.01, 1.04) | 1.04 (1.02, 1.06) | 1.02 (1.00, 1.04) | 1.04 (1.03, 1.06) |
|  | 96th | 1.07 (1.03, 1.11) | 1.04 (1.02, 1.05) | 1.03 (1.02, 1.05) | 1.05 (1.03, 1.08) | 1.02 (1.00, 1.05) | 1.05 (1.03, 1.07) |
|  | 97th | 1.09 (1.04, 1.14) | 1.04 (1.02, 1.07) | 1.04 (1.02, 1.06) | 1.07 (1.04, 1.10) | 1.03 (1.00, 1.06) | 1.06 (1.04, 1.09) |
|  | 98th | 1.12 (1.05, 1.19) | 1.06 (1.02, 1.10) | 1.05 (1.01, 1.09) | 1.10 (1.05, 1.15) | 1.04 (1.01, 1.08) | 1.08 (1.04, 1.13) |
|  | 99th | 1.18 (1.06, 1.31) | 1.08 (1.01, 1.16) | 1.06 (0.99, 1.14) | 1.16 (1.07, 1.25) | 1.07 (1.00, 1.15) | 1.12 (1.04, 1.21) |
|  | 99.9th | 1.31 (1.06, 1.63) | 1.14 (0.96, 1.34) | 1.09 (0.93, 1.28) | 1.30 (1.10, 1.55) | 1.13 (0.97, 1.33) | 1.19 (1.00, 1.42) |
|  | P value | 0.204 | | 0.414 | | 0.26 | |
| Lag 9 | 95th | 1.04 (1.01, 1.07) | 1.03 (1.01, 1.05) | 1.02 (1.01, 1.04) | 1.03 (1.02, 1.05) | 1.01 (0.99, 1.03) | 1.04 (1.02, 1.06) |
|  | 96th | 1.06 (1.02, 1.09) | 1.04 (1.02, 1.06) | 1.03 (1.01, 1.05) | 1.05 (1.02, 1.07) | 1.02 (0.99, 1.04) | 1.05 (1.03, 1.07) |
|  | 97th | 1.07 (1.03, 1.12) | 1.04 (1.02, 1.07) | 1.04 (1.01, 1.06) | 1.06 (1.03, 1.10) | 1.03 (1.00, 1.05) | 1.06 (1.03, 1.09) |
|  | 98th | 1.10 (1.04, 1.17) | 1.05 (1.01, 1.10) | 1.05 (1.01, 1.09) | 1.09 (1.04, 1.14) | 1.04 (1.00, 1.08) | 1.08 (1.03, 1.12) |
|  | 99th | 1.16 (1.05, 1.28) | 1.08 (0.99, 1.17) | 1.06 (0.99, 1.14) | 1.15 (1.06, 1.25) | 1.07 (1.00, 1.16) | 1.11 (1.03, 1.20) |
|  | 99.9th | 1.30 (1.06, 1.59) | 1.12 (0.93, 1.35) | 1.10 (0.93, 1.30) | 1.29 (1.07, 1.56) | 1.16 (0.97, 1.38) | 1.18 (0.99, 1.41) |
|  | P value | 0.486 | | 0.477 | | 0.207 | |
| Lag 10 | 95th | 1.05 (1.02, 1.08) | 1.03 (1.01, 1.04) | 1.03 (1.01, 1.04) | 1.04 (1.02, 1.06) | 1.01 (0.99, 1.04) | 1.04 (1.02, 1.06) |
|  | 96th | 1.06 (1.03, 1.10) | 1.03 (1.01, 1.05) | 1.03 (1.01, 1.05) | 1.05 (1.02, 1.07) | 1.02 (1.00, 1.04) | 1.05 (1.03, 1.07) |
|  | 97th | 1.08 (1.03, 1.12) | 1.04 (1.01, 1.07) | 1.04 (1.01, 1.07) | 1.06 (1.03, 1.09) | 1.03 (1.00, 1.06) | 1.06 (1.03, 1.09) |
|  | 98th | 1.10 (1.04, 1.17) | 1.05 (1.00, 1.09) | 1.05 (1.00, 1.09) | 1.08 (1.04, 1.13) | 1.04 (1.00, 1.08) | 1.08 (1.03, 1.12) |
|  | 99th | 1.14 (1.03, 1.26) | 1.07 (0.98, 1.16) | 1.06 (0.98, 1.14) | 1.13 (1.05, 1.23) | 1.06 (0.98, 1.15) | 1.10 (1.02, 1.19) |
|  | 99.9th | 1.23 (1.00, 1.51) | 1.11 (0.91, 1.34) | 1.08 (0.91, 1.29) | 1.25 (1.05, 1.50) | 1.12 (0.92, 1.36) | 1.16 (0.97, 1.38) |
|  | P value | 0.571 | | 0.720 | | 0.187 | |

-65: below 65 years old, +65: 65 or more years old, CRR: cumulative relative risk, CI: confidence interval. CRRs were calculated through meta-analysis of coefficients estimated by distributed lag nonlinear models according to the province. CRRs are relative values that compare the mortality risks at 33℃. Each period covers June to September per year. Daily maximum temperatures are the average of 95th, 96th, 97th, 98th, 99th, and 99.9th percentile for each region across the two periods.

**Test for the Difference Between 1991-1995 and 2015-2019 According to Maximum Lagged Effect**

|  |  | Nonlinear relationship | CRR at 99th percentile |
| --- | --- | --- | --- |
|  |  | (cross-basis coefficients) |  |
| Maximum-lagged effect | Population | P-value for the difference between 1991-1995 and 2015-2019 | |
| **lag 3** | Overall population | 0.045 | 0.226 |
|  | Male, -65 | 0.312 | 0.420 |
|  | Female, -65 | 0.837 | 0.575 |
|  | Male, +65 | 0.170 | 0.269 |
|  | Female, +65 | 0.263 | 0.076 |
| **lag 4** | Overall population | 0.170 | 0.241 |
|  | Male, -65 | 0.322 | 0.189 |
|  | Female, -65 | 0.956 | 0.714 |
|  | Male, +65 | 0.475 | 0.401 |
|  | Female, +65 | 0.284 | 0.120 |
| **lag 5** | Overall population | 0.394 | 0.215 |
|  | Male, -65 | 0.168 | 0.084 |
|  | Female, -65 | 0.974 | 0.833 |
|  | Male, +65 | 0.753 | 0.510 |
|  | Female, +65 | 0.087 | 0.128 |
| **lag 7** | Overall population | 0.290 | 0.193 |
|  | Male, -65 | 0.258 | 0.227 |
|  | Female, -65 | 0.532 | 0.252 |
|  | Male, +65 | 0.399 | 0.421 |
|  | Female, +65 | 0.281 | 0.185 |
| **lag 8** | Overall population | 0.204 | 0.197 |
|  | Male, -65 | 0.169 | 0.270 |
|  | Female, -65 | 0.618 | 0.370 |
|  | Male, +65 | 0.182 | 0.652 |
|  | Female, +65 | 0.515 | 0.217 |
| **lag 9** | Overall population | 0.486 | 0.256 |
|  | Male, -65 | 0.503 | 0.587 |
|  | Female, -65 | 0.288 | 0.189 |
|  | Male, +65 | 0.252 | 0.968 |
|  | Female, +65 | 0.663 | 0.241 |
| **lag 10** | Overall population | 0.571 | 0.322 |
|  | Male, -65 | 0.538 | 0.759 |
|  | Female, -65 | 0.384 | 0.134 |
|  | Male, +65 | 0.231 | 0.830 |
|  | Female, +65 | 0.260 | 0.250 |

**2. Sensitivity analysis for other cross-basis parameters**

**Knots for exposure-response function**

We conducted a sensitivity analysis by modifying the internal knots of the exposure basis parameters to the 25th and 75th percentiles.

**CRR on mortality of high daily maximum temperature for summer by period, gender and age group**

| Daily maximum temperatures | Study periods | | Gender | | Age | |
| --- | --- | --- | --- | --- | --- | --- |
|  | 1991–1995 | 2015–2019 | Male | Female | -65 | +65 |
|  | CRR (95% CI) | | | | | |
| 90th | 1.01 (1.00, 1.01) | 1.01 (1.00, 1.01) | 1.00 (1.00, 1.01) | 1.01 (1.01, 1.01) | 1.00 (1.00, 1.01) | 1.01 (1.00, 1.01) |
| 91th | 1.02 (1.01, 1.03) | 1.01 (1.00, 1.01) | 1.01 (1.00, 1.01) | 1.01 (1.01, 1.02) | 1.01 (1.00, 1.01) | 1.01 (1.01, 1.02) |
| 92th | 1.02 (1.01, 1.04) | 1.01 (1.01, 1.02) | 1.01 (1.00, 1.02) | 1.02 (1.01, 1.03) | 1.01 (1.00, 1.02) | 1.02 (1.01, 1.03) |
| 93th | 1.03 (1.01, 1.05) | 1.02 (1.01, 1.03) | 1.02 (1.00, 1.03) | 1.03 (1.02, 1.04) | 1.01 (1.00, 1.02) | 1.03 (1.01, 1.04) |
| 94th | 1.04 (1.01, 1.06) | 1.02 (1.01, 1.04) | 1.02 (1.01, 1.03) | 1.04 (1.02, 1.05) | 1.01 (1.00, 1.03) | 1.03 (1.02, 1.05) |
| 95th | 1.05 (1.02, 1.08) | 1.03 (1.01, 1.05) | 1.02 (1.00, 1.04) | 1.05 (1.03, 1.07) | 1.02 (1.00, 1.03) | 1.04 (1.02, 1.07) |
| 96th | 1.06 (1.02, 1.10) | 1.04 (1.01, 1.06) | 1.03 (1.00, 1.05) | 1.06 (1.03, 1.09) | 1.02 (1.00, 1.04) | 1.05 (1.03, 1.08) |
| 97th | 1.07 (1.02, 1.13) | 1.05 (1.01, 1.08) | 1.03 (1.00, 1.07) | 1.08 (1.04, 1.11) | 1.02 (1.00, 1.05) | 1.07 (1.03, 1.10) |
| 98th | 1.09 (1.02, 1.17) | 1.06 (1.01, 1.11) | 1.04 (1.00, 1.09) | 1.10 (1.05, 1.15) | 1.03 (0.99, 1.07) | 1.09 (1.04, 1.14) |
| 99th | 1.13 (1.02, 1.25) | 1.09 (1.01, 1.16) | 1.06 (0.99, 1.13) | 1.14 (1.07, 1.23) | 1.04 (0.98, 1.10) | 1.12 (1.04, 1.20) |
| 99.9th | 1.21 (1.02, 1.43) | 1.14 (1.01, 1.29) | 1.08 (0.96, 1.22) | 1.24 (1.10, 1.40) | 1.06 (0.95, 1.18) | 1.20 (1.06, 1.35) |
| P value | 0.216 | | 0.442 | | 0.090 | |

-65: below 65 years old, +65: 65 or more years old, CRR: cumulative relative risk, CI: confidence interval. CRRs were calculated through meta-analysis of coefficients estimated by distributed lag nonlinear models according to the province. CRRs are relative values that compare the mortality risks at 33℃. Each period covers June to September per year. Daily maximum temperatures are the average of 90th, 91th, 92th, 93th, 94th, 95th, 96th, 97th, 98th, 99th, and 99.9th percentile for each region across the two periods.

**Test for the difference between 1991-1995 and 2015-2019**

|  | Nonlinear relationship | CRR at 99th percentile |
| --- | --- | --- |
|  | (cross-basis coefficients) |  |
| Population | P-value for the difference between 1991-1995 and 2015-2019 | |
| Overall population | 0.216 | 0.510 |
| Male, -65 | 0.262 | 0.494 |
| Female, -65 | 0.807 | 0.952 |
| Male, +65 | 0.509 | 0.718 |
| Female, +65 | 0.115 | 0.243 |

-65: below 65 years old, +65: 65 or more years old. Differences in the nonlinear relationships were tested using the multivariate Wald test. Differences in CRR at 99th percentile, centered at 33℃, were tested using Z-test

**Knots for lag-response function**

We conducted a sensitivity analysis by modifying the number of internal knots in the lag basis parameters to 3.

**CRR on mortality of high daily maximum temperature for summer by period, gender and age group**

| Daily maximum temperatures | Study periods | | Gender | | Age | |
| --- | --- | --- | --- | --- | --- | --- |
|  | 1991–1995 | 2015–2019 | Male | Female | -65 | +65 |
|  | CRR (95% CI) | | | | | |
| 90th | 1.01 (1.00, 1.01) | 1.00 (1.00, 1.01) | 1.01 (1.00, 1.01) | 1.01 (1.00, 1.01) | 1.00 (1.00, 1.01) | 1.01 (1.00, 1.01) |
| 91th | 1.02 (1.01, 1.03) | 1.01 (1.00, 1.01) | 1.01 (1.00, 1.02) | 1.01 (1.00, 1.02) | 1.01 (1.00, 1.01) | 1.01 (1.01, 1.02) |
| 92th | 1.02 (1.01, 1.04) | 1.01 (1.00, 1.02) | 1.01 (1.00, 1.02) | 1.02 (1.01, 1.03) | 1.01 (1.00, 1.02) | 1.02 (1.01, 1.03) |
| 93th | 1.03 (1.02, 1.05) | 1.02 (1.01, 1.03) | 1.02 (1.01, 1.03) | 1.02 (1.01, 1.03) | 1.01 (1.00, 1.02) | 1.03 (1.02, 1.04) |
| 94th | 1.04 (1.02, 1.06) | 1.02 (1.01, 1.03) | 1.02 (1.01, 1.03) | 1.03 (1.02, 1.04) | 1.01 (1.00, 1.03) | 1.03 (1.02, 1.04) |
| 95th | 1.05 (1.03, 1.08) | 1.03 (1.01, 1.04) | 1.03 (1.01, 1.04) | 1.04 (1.02, 1.06) | 1.02 (1.00, 1.03) | 1.04 (1.03, 1.06) |
| 96th | 1.07 (1.03, 1.10) | 1.03 (1.02, 1.05) | 1.03 (1.02, 1.05) | 1.05 (1.03, 1.08) | 1.02 (1.00, 1.04) | 1.05 (1.04, 1.07) |
| 97th | 1.08 (1.04, 1.13) | 1.04 (1.02, 1.07) | 1.04 (1.02, 1.06) | 1.07 (1.04, 1.10) | 1.03 (1.00, 1.05) | 1.07 (1.04, 1.09) |
| 98th | 1.11 (1.05, 1.18) | 1.06 (1.02, 1.10) | 1.05 (1.02, 1.09) | 1.10 (1.05, 1.14) | 1.03 (1.00, 1.07) | 1.09 (1.05, 1.13) |
| 99th | 1.16 (1.06, 1.27) | 1.09 (1.01, 1.17) | 1.07 (1.00, 1.13) | 1.15 (1.07, 1.24) | 1.05 (0.99, 1.11) | 1.13 (1.05, 1.22) |
| 99.9th | 1.27 (1.05, 1.53) | 1.15 (0.98, 1.35) | 1.10 (0.96, 1.26) | 1.28 (1.09, 1.50) | 1.08 (0.94, 1.24) | 1.22 (1.03, 1.44) |
| P value | 0.463 | | 0.520 | | 0.063 | |

-65: below 65 years old, +65: 65 or more years old, CRR: cumulative relative risk, CI: confidence interval. CRRs were calculated through meta-analysis of coefficients estimated by distributed lag nonlinear models according to the province. CRRs are relative values that compare the mortality risks at 33℃. Each period covers June to September per year. Daily maximum temperatures are the average of 90th, 91th, 92th, 93th, 94th, 95th, 96th, 97th, 98th, 99th, and 99.9th percentile for each region across the two periods.

**Test for the difference between 1991-1995 and 2015-2019**

|  | Nonlinear relationship | CRR at 99th percentile |
| --- | --- | --- |
|  | (cross-basis coefficients) |  |
| Population | P-value for the difference between 1991-1995 and 2015-2019 | |
| Overall population | 0.463 | 0.274 |
| Male, -65 | 0.369 | 0.255 |
| Female, -65 | 0.812 | 0.677 |
| Male, +65 | 0.594 | 0.488 |
| Female, +65 | 0.079 | 0.189 |

-65: below 65 years old, +65: 65 or more years old. Differences in the nonlinear relationships were tested using the multivariate Wald test. Differences in CRR at 99th percentile, centered at 33℃, were tested using Z-test

**3. Sensitivity analysis using the average daily maximum temperature of stations by Province**

Instead of using the closest measurement station to the center of each province, we conducted a sensitivity analysis using the average daily maximum temperature from all measurement stations within the province.

**Number of weather stations by province and period and Correlation between two metrics**

The number of weather stations per province for each period is as follows. Correlation between two exposure metrics (the daily maximum temperature of the closest measurement station to the center of each province vs. the average daily maximum temperature of all measurement stations within the province) was 0.94.

| **Province** | **1991-1995** | **2015-2019** |
| --- | --- | --- |
| Seoul | 1 | 1 |
| Busan | 1 | 1 |
| Daegu | 1 | 2 |
| Incheon | 2 | 3 |
| Gwngju | 1 | 1 |
| Daejeon | 1 | 1 |
| Gyeonggi-do | 3 | 5 |
| Gangwon-do | 12 | 14 |
| Chungcheongbuk-do | 5 | 5 |
| Chungcheongnam-do | 5 | 6 |
| Jeollabuk-do | 7 | 10 |
| Jeollanam-do | 8 | 14 |
| Gyeongsangbuk-do | 11 | 14 |
| Gyeonsangnam-do | 9 | 14 |
| Jeju-do | 6 | 4 |

**CRR on mortality of high daily maximum temperature for summer by period, gender and age group (using the average daily maximum temperature of all measurement stations within the province)**

In the overall population, the p-value for the difference in cross-basis coefficients between thetwo periods was 0.058, which is not statistically significant at the 0.05 level. However, a decreasing trend in CRR in the recent period was observed. The CRR was higher in the population over 65 compared to those under 65, with a statistically significant difference (p-value = 0.018).

|  | **Study periods** | | **Gender** | | **Age** | |
| --- | --- | --- | --- | --- | --- | --- |
|  | **1991–1995** | **2015–2019** | **Male** | **Female** | **-65** | **+65** |
| **Daily maximum temperatures** | CRR (95% CI) | CRR (95% CI) | CRR (95% CI) | CRR (95% CI) | CRR (95% CI) | CRR (95% CI) |
| 32.9℃ | 1.00 (0.99, 1.00) | 1.00 (1.00, 1.00) | 1.00 (1.00, 1.00) | 1.00 (1.00, 1.00) | 1.00 (1.00, 1.00) | 1.00 (1.00, 1.00) |
| 33.1℃ | 1.00 (1.00, 1.01) | 1.00 (1.00, 1.00) | 1.00 (1.00, 1.00) | 1.00 (1.00, 1.00) | 1.00 (1.00, 1.00) | 1.00 (1.00, 1.00) |
| 33.3℃ | 1.01 (1.01, 1.02) | 1.01 (1.00, 1.01) | 1.01 (1.00, 1.01) | 1.01 (1.01, 1.01) | 1.01 (1.00, 1.01) | 1.01 (1.01, 1.01) |
| 33.6℃ | 1.02 (1.02, 1.03) | 1.01 (1.00, 1.02) | 1.01 (1.01, 1.02) | 1.02 (1.01, 1.02) | 1.01 (1.00, 1.02) | 1.02 (1.01, 1.02) |
| 33.8℃ | 1.04 (1.02, 1.05) | 1.02 (1.01, 1.02) | 1.02 (1.01, 1.03) | 1.03 (1.02, 1.03) | 1.01 (1.00, 1.02) | 1.03 (1.02, 1.03) |
| 34.1℃ | 1.05 (1.04, 1.07) | 1.02 (1.01, 1.03) | 1.02 (1.01, 1.03) | 1.04 (1.02, 1.05) | 1.02 (1.01, 1.03) | 1.04 (1.03, 1.05) |
| 34.4℃ | 1.07 (1.05, 1.09) | 1.03 (1.02, 1.05) | 1.03 (1.02, 1.05) | 1.05 (1.03, 1.07) | 1.02 (1.01, 1.04) | 1.05 (1.03, 1.06) |
| 34.7℃ | 1.09 (1.06, 1.12) | 1.04 (1.02, 1.07) | 1.04 (1.02, 1.06) | 1.07 (1.04, 1.09) | 1.03 (1.01, 1.05) | 1.06 (1.04, 1.09) |
| 35.2℃ | 1.13 (1.09, 1.18) | 1.06 (1.02, 1.10) | 1.05 (1.02, 1.09) | 1.09 (1.06, 1.13) | 1.04 (1.01, 1.07) | 1.09 (1.05, 1.13) |
| 35.9℃ | 1.20 (1.12, 1.28) | 1.10 (1.02, 1.18) | 1.08 (1.02, 1.15) | 1.15 (1.08, 1.22) | 1.06 (1.00, 1.12) | 1.13 (1.06, 1.21) |
| 37.2℃ | 1.37 (1.20, 1.56) | 1.19 (1.02, 1.39) | 1.13 (1.00, 1.29) | 1.28 (1.12, 1.46) | 1.10 (0.96, 1.25) | 1.24 (1.07, 1.44) |
| *P* value | 0.058 | | 0.586 | | 0.018 | |

-65: below 65 years old, +65: 65 or more years old, CRR: cumulative relative risk, CI: confidence interval. CRRs were calculated through meta-analysis of coefficients estimated by distributed lag nonlinear models according to the province. CRRs are relative values that compare the mortality risks at 33℃. Each period covers June to September per year. Daily maximum temperatures are the average of 90th, 91th, 92th, 93th, 94th, 95th, 96th, 97th, 98th, 99th, and 99.9th percentile for each region across the two periods.

**Nonlinear association between daily maximum temperature and mortality by period, gender and age group (using the average daily maximum temperature of all measurement stations within the province)**


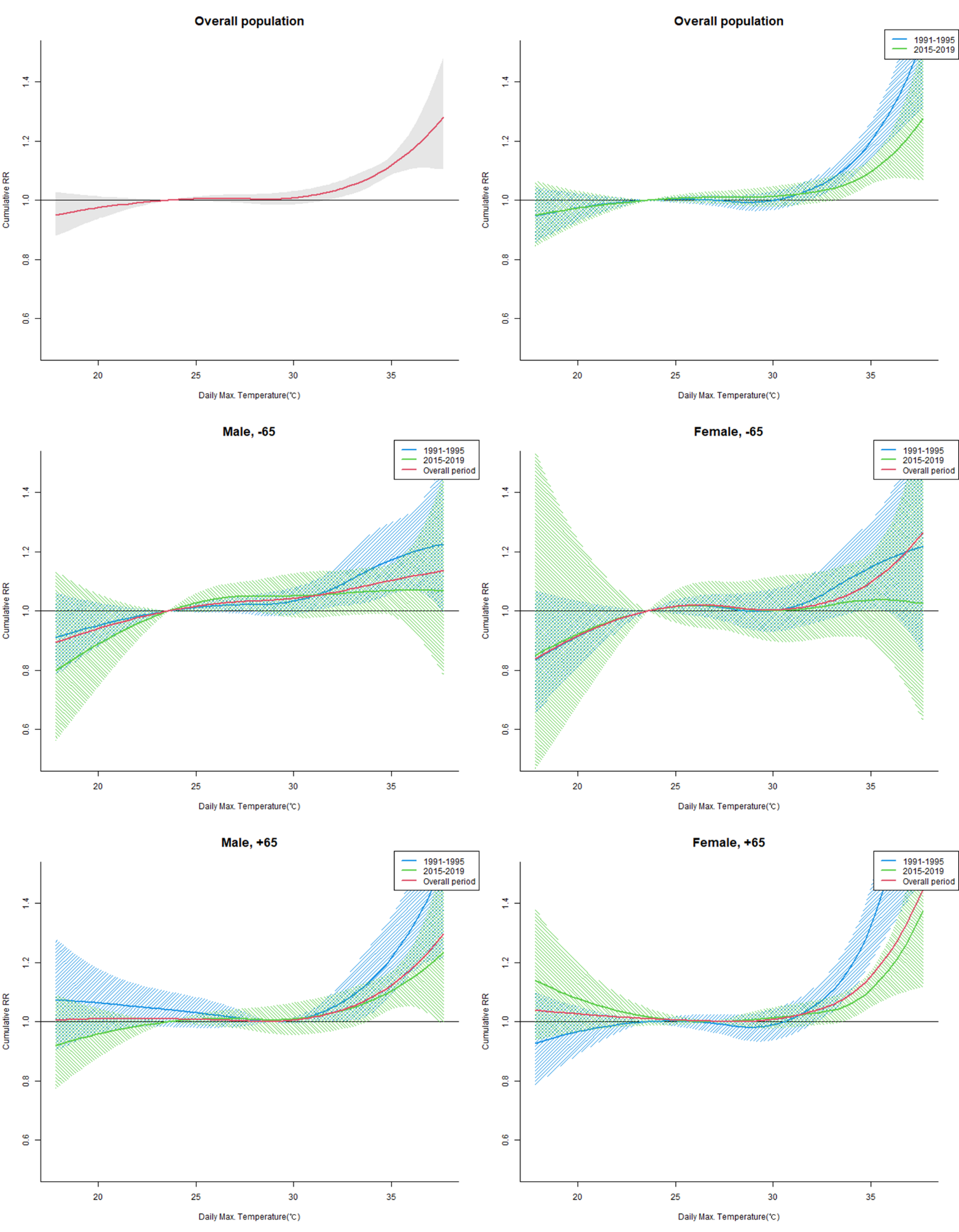


Max.: maximum, -65: below 65 years old, +65: 65 or more years old. Each period covers June to September per year. The relationships between summer daily maximum temperature and mortality were determined through meta-analysis of coefficients estimated by distributed lag nonlinear models by province. The CRR in the plots is centered on the MMT (If the MMT is less than the 10th percentile, the 10th percentile was used as centering value). The red line represents the relationship over the entire period. Blue lines represent relationships in the period 1991-1995, and green lines represent relationships in the period 2015-2019. The shaded area around the line means the 95% confidence interval.

**Test for the difference between 1991-1995 and 2015-2019 (using the average daily maximum temperature of all measurement stations within the province)**

When testing the differences between the two periods by subpopulation, the differences in nonlinear relationship and in CRR at the 99th percentile were statistically significant in females over 65.

|  | Nonlinear relationship (cross-basis coefficients) | CRR at 99th percentile |
| --- | --- | --- |
|  | P-value for the difference between 1991-1995 and 2015-2019 | |
| Overall population | 0.058 | 0.078 |
| Male, -65 | 0.394 | 0.354 |
| Female, -65 | 0.832 | 0.526 |
| Male, +65 | 0.311 | 0.158 |
| Female, +65 | 0.003 | 0.019 |

-65: below 65 years old, +65: 65 or more years old. Differences in the nonlinear relationships were tested using the multivariate Wald test. Differences in CRR at 99th percentile, centered at 33℃, were tested using Z-test

**Impacts of high air temperature of summer in Korea (using the average daily maximum temperature of all measurement stations within the province)**

In the analysis using the average daily maximum temperature per province, the impacts of high air temperature during summer are as follows.

|  | **Past** | | **Recent**  **(Aging case)** | | **Non-aging scenario** | |
| --- | --- | --- | --- | --- | --- | --- |
|  | **1991–1995** | **1994** | **2015–2019** | **2018** | **2015–2019** | **2018** |
| Person-year | 72,151,527 | 14,566,888 | 83,266,268 | 16,716,190 | 83,266,268 | 16,716,190 |
| Observed death counts | 375,810 | 78,884 | 440,553 | 91,249 | 250,403 | 51,123 |
| Excess death counts (95% eCIs) | 2,752 (1,640, 3,450) | 2,452 (1,386, 3,109) | 2,308 (399, 3,769) | 1,467 (166, 2,263) | 1,084 | 673 |
| AF (95% eCIs) (%) | 0.7 (0.4, 0.9) | 3.1 (1.8, 3.9) | 0.5 (0.1, 0.9) | 1.6 (0.2, 2.5) | 0.4 | 1.3 |
| Excess death rate (95% eCIs) (/100,000PY) | 3.8 (2.3, 4.8) | 16.8 (9.5, 21.3) | 2.8 (0.5, 4.5) | 8.8 (1.0, 13.5) | 1.3 | 4.0 |
| AF: Attributable fraction, eCIs: empirical confidence Intervals, PY: person-years. Each period covers June to September per year The non-aging scenario assumes that the gender-age distribution of person-years in recent periods was the same as that in past periods and applied the stratum-specific excess death rate. Observed death counts in the non-aging scenario were calculated by assuming the past gender-age distribution in the recent person-years. | | | | | | |
